# Carotid Plaque Composition and the Importance of Non-Invasive in Imaging Stroke Prevention

**DOI:** 10.3389/fcvm.2022.885483

**Published:** 2022-05-16

**Authors:** Martin Andreas Geiger, Ronald Luiz Gomes Flumignan, Marcone Lima Sobreira, Wagner Mauad Avelar, Carla Fingerhut, Sokrates Stein, Ana Terezinha Guillaumon

**Affiliations:** ^1^Division of Vascular Surgery, Department of Surgery, Universidade Estadual de Campinas—UNICAMP, São Paulo, Brazil; ^2^Division of Vascular and Endovascular Surgery, Department of Surgery, Universidade Federal de São Paulo, São Paulo, Brazil; ^3^Division of Vascular and Endovascular Surgery, Department of Surgery and Orthopedics, Botucatu Medical School, Universidade Estadual Paulista (UNESP), São Paulo, Brazil; ^4^Department of Neurology, Universidade Estadual de Campinas—UNICAMP, São Paulo, Brazil; ^5^Division of Radiology, Department of Anesthesiology and Radiology, Universidade Estadual de Campinas—UNICAMP, São Paulo, Brazil

**Keywords:** ICA stenosis, ischemic stroke, vulnerable plaque biomarker, MRI, Atherosclerosis

## Abstract

Luminal stenosis has been the standard feature for the current management strategies in patients with atherosclerotic carotid disease. Histological and imaging studies show considerable differences between plaques with identical degrees of stenosis. They indicate that specific plaque characteristics like Intraplaque hemorrhage, Lipid Rich Necrotic Core, Plaque Inflammation, Thickness and Ulceration are responsible for the increased risk of ischemic events. Intraplaque hemorrhage is defined by the accumulation of blood components within the plaque, Lipid Rich Necrotic Core is composed of macrophages loaded with lipid, Plaque Inflammation is defined as the process of atherosclerosis itself and Plaque thickness and Ulceration are defined as morphological features. Advances in imaging methods like Magnetic Resonance Imaging, Ultrasound, Computed Tomography and Positron Emission Tomography have enabled a more detailed characterization of the plaque, and its vulnerability is linked to these characteristics, changing the management of these patients based only on the degree of plaque stenosis. Studies like Rotterdam, ARIC, PARISK, CAPIAS and BIOVASC were essential to evaluate and prove the relevance of these characteristics with cerebrovascular symptoms. A better approach for the prevention of stroke is needed. This review summarizes the more frequent carotid plaque features and the available validation from recent studies with the latest evidence.

## Introduction

Stroke is the second leading cause of death worldwide. More than 12 million people have a stroke annually, of which more than 6 million die as a result of the event (1–3). The atherosclerotic carotid disease accounts for 10–15% of stroke and transient ischemic attack (TIA) cases. It usually occurs at the carotid bifurcation and internal carotid artery (ICA) (1). Vessel stenosis is the main parameter for classifying and stratifying the disease and is adopted by the main guidelines to determine surgical intervention. However, recent evidence suggests that specific plaque features may be more directly associated with stroke than just stenosis. The study of the vessel and plaque is the current target of several researchers. This change of focus reveals its importance for primary and secondary stroke prevention (4).

These observations were previously made in the coronary territory where non-contrast cardiac computed tomography is routinely performed for risk stratification in primary prevention, to quantify coronary artery calcification as an imaging test of subclinical atherosclerosis. The assessment of coronary inflammation represents, as in carotid disease, a new aspect in the assessment of coronary artery disease resulting in an improvement in the prediction, discrimination and reclassification of all causes and cardiac mortality. Analysis of plaque extent, plaque composition, and inflammation has the potential to establish a more accurate risk prediction compared to coronary calcium score assessed by non-contrast cardiac CT (5).

In the first part of this review, we recall the current features of carotid plaques vulnerability, the best imaging methods, and its characteristics for the assessment. In the second part, we discuss the predictive value of plaque imaging in primary and secondary prevention, linking plaque characteristics and their role in clinical decision-making.

## Plaque Characteristics

### Intraplaque Hemorrhage

**Figure 1:**
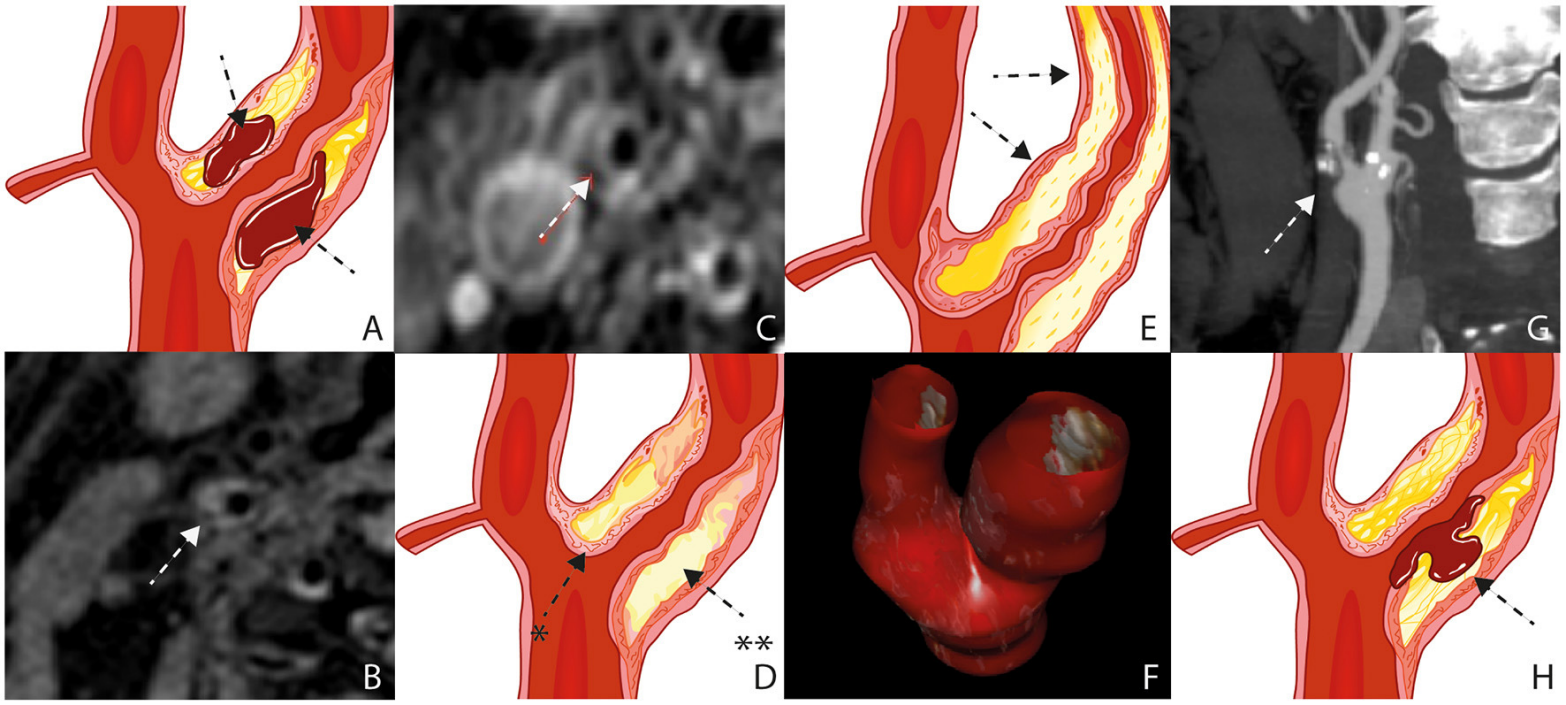
Plaque Characteristics. Schematic figure illustrating intraplaque hemorrhage **(A)**, MRI on a 3 Tesla scanner **(B)**; white arrow), The axial T1 turbo spin-echo with fat saturation image post gadolinium shows a pronounced LRNC covered by an intact fibrous cap **(C)**; white arrow), Schematic figure illustrating a pronounced LRNC (^**^) covered by an intact fibrous cap (^*^) **(D)**, Schematic figure illustrating a pronounced bulky plaque **(E)**; black arrows), 3D ultrasound volume analysis **(F)**, surface morphology, image obtained by CT **(G)**, Schematic figure illustrating plaque rupture with an ulceration **(H)**; black arrow). LRNC, lipid-rich necrotic core; MRI, Magnetic Resonance Imaging; CT,Computed Tomography.

Intraplaque hemorrhage (IPH) is considered a major predictor of patient symptoms from carotid plaque (Figures 1A,B) (6). The atheroma neovascularization is immature (7). There is a difference observed between the neovessels in the external part of the media and the neovessels which reach the plaque (8). The development and maturation of normal neovascularisation seems to be impaired by a proteolytic environment within the plaque (9). IPH is the fundamental process for plaque growth in more advanced atherosclerotic plaque stages (10).

In a recent systematic review, the hazard ratio (HR) of 7.14 was the highest in the presence of IPH in recurrent Stroke/Transient Ischemic Attack (TIA) compared to other plaque characteristics such as lipid-rich necrotic core and fibrous cap rupture (11). In another systematic review focusing on stenosis <50% in patients with embolic stroke of undetermined cause, the prevalence of IPH in the ipsilateral carotid was 24.4% (95% CI 17.9 to 31.5) compared to 0.6% (95% CI 0.0 to 3.7) in the contralateral carotid (12).

Schindler *et al*. showed in a recent published meta-analysis that IPH increased the risk of future ipsilateral stroke in patients with symptomatic and asymptomatic carotid stenosis, with HRs of 10.2 and 7.9, respectively (13).

Magnetic resonance imaging (MRI) is the best imaging technique to atherosclerotic plaque characterization, specially IPH (Figure 1B) (14–16). Computed tomography (CT) can overestimate the disease in heavily calcified lesions, besides being difficult to differentiate between fibrous, lipid, and intraplaque hemorrhage (17).

### Fibrous Cap and Lipid-Rich Necrotic Core

The fibrous cap (FC) separates the Lipid Rich Necrotic Core (LRNC) from the vessel lumen (Figures 1C,D). It is composed of muscle cells, macrophages, foam cells, lymphocytes, collagen, and elastin (18). FC rupture exposes the necrotic core to blood resulting in cerebral microembolization (19).

In vulnerable plaques, it is observed that a thin FC enfolds a large LRNC containing mainly inflammatory cells and macrophages (20).

### Plaque Inflammation/ Neovascularisation

Inflammation is a pathogenic event characterized by several structural changes in the vessel wall. In this process, fundamental steps are observed like endothelial dysfunction, macrophage activation and migration, oxidative stress, lipid deposition, proliferation and migration of smooth muscle cells and formation of neovessels within the plaque. (7) Neovascularization increases the local flow of nutrients and O2 promoting plaque growth. The incomplete maturation of these neovessels associated with fragility promotes IPH leaving the plaque vulnerable and prone to rupture (21).

Contrast-enhanced ultrasound (CEUS) allows an objective analysis of atherosclerotic plaque inflammation (Additional File) (22). In the early phase, after contrast administration, the neovessels of the atherosclerotic plaque are filled with blood, and the intact microspheres amplify their echo. In the late phase of contrast injection, juxtaluminal black areas (JBA) can be observed and distinguished, which are very hypoechoic areas without a fibrous cap with fragments of lipid core in ruptured plaques with echogenicity lower than 25 gray-scale median (GSM) units (Additional File) (23).

Previous studies using either CEUS or dynamic contrast-enhanced magnetic resonance imaging have demonstrated associations between imaging measurements of neovessels and recent symptoms. Both methods are validated non-invasive techniques for *in vivo* imaging of neo-vessels (24, 25). Van den Oord *et al*. estimated the Cardiovascular risk of recruited patients by calculating the Prospective Cardiovascular Münster Heart Study (PROCAM) risk. Carotid CEUS was performed in these patients. Interestingly, CEUS changed the risk category in practically all asymptomatic patients previously classified by the traditional risk stratification model (26).

MRI has demonstrated its importance in the detailed characterization of the carotid plaque. Other imaging methods are being used to search for more careful definition. ^18^F-fluorodeoxyglucose (FDG) positron emission tomography (PET) may be an alternative technique to identify a plaque with inflammation (27). Tawakol *et al*. firstly found histological association of plaque inflammation with the degree of ^18^F-FDG uptake (28). The abundance of inflammatory cells is observed in the highly inflamed vulnerable plaque. FDG is a glucose analog taken up by inflammatory cells. A more significant accumulation of FDG is observed with higher metabolic activity (27). More recently, another commonly employed radiotracer, NaF, is being used due to its affinity to hydroxyapatite. More extensive calcium deposits can be easily observed with CT; however, micro-calcifications are not. NaF accumulates in areas of active micro-calcification within the atheroma. An interesting feature is that ^18^F-NaF uptake does not overlap with macrocalcifications as seen on CT (29).

Skagen *et al*. demonstrated that ^18^F-FDG uptake on PET/CT was higher in patients with symptomatic compared with asymptomatic carotid artery plaques (30).

Fujimoto *et al*. in a recent publication demonstrated that 18F Sodium Fluoride (^18^F-NaF) uptake was associated with the severity of ischemic vascular brain disease on MRI, suggesting its possible use in the risk classification of cerebrovascular disease (31).

### Carotid Plaque Thickness

The plaque thickness is a feature of plaque vulnerability being associated with the size and volume of the plaque (Figure 1E) (32). It is well quantifiable with ultrasound, CT, and MRI. Zhao X *et al*. observed that wall thickness was found to be a stronger feature when compared to stenosis for high risk carotid plaques, already observed and published by the same author in 2011 analyzing stenosis, percentage of wall volume (PWV) and mean wall thickness (33). The possible explanation is positive remodeling of the plaque. The outward expansion of the outer wall boundary would preserve the vessel lumen. In other words, wall thickness may be an important feature for screening of high-risk plaques composition (34).

Recently, Ball S. *et al*. published a study using a technology called tomographic ultrasound (tUS) (Figure 1F) (35). It consist of a three-dimensional (3D) ultrasound system with a spacial tracker (Piur, Wien, Austria) Multiplanar reconstructions are computed to produce 3D ultrasound volumes. It showed to be an accurate method with all the advantages of ultrasound.

### Carotid Plaque Surface (Ulceration)

High-risk carotid plaques are not only characterized by its compositional features but also by the irregularity of the plaque surface (36). Ulceration is defined as a discontinuous fibrous cap with an excavated necrotic core. (Figures 1G,H) (8) Jin Li et al. found a direct association between irregular plaque surface and carotid plaque features, such as LRNC, IPH, stenosis, and maximum wall thickness (37).

Homburg et al. demonstrated an association between plaque composition and volume with plaque ulceration and ischemic stroke patients independently of the degree of the carotid plaque stenosis (38).

IPH, apparently, increase inflammation resulting in irregular plaque surface. Hamada et al. validated plaque ulceration assessed by CEUS with histology analysis in patients undergoing carotid endarterectomy confirming CEUS's high sensitivity for identifying plaque ulceration and fibrous cap disruption (39).

## Discussion

### Prediction of Primary Stroke

The occurrence of a carotid plaque rupture is independent of the degree of stenosis and the plaque features described above are related to (40).

In a recent meta-analysis of 64 studies enrolling 20,751 asymptomatic participants, the authors observed that the incidence of ipsilateral ischemic events was higher in patients with high-risk features than in those without high-risk features with a corresponding OR of 3.0. (12). In another meta-analysis of 8 studies, the presence of IPH at baseline was associated with a 6-fold higher risk of cerebrovascular events, with an annualized event rate of 17.7% compared with 2.43% in patients with no IPH (41). Schindler *et al.*, comparing the risk of stroke between patients with and without the presence of IPH on MRI, observed 5.4% event rates among patients with asymptomatic carotid stenosis in those with IPH vs. 0.8% in those without IPH (13). These facts suggest that carotid IPH might be a fundamental biomarker of clinical ischemic events.

IPH is detected as a high signal intensity on T1-weighted imaging. In a study with 1,190 patients, carotid T1-high-intense plaque was at higher risk of a subsequent cerebrovascular ischemic event suggesting that carotid IPH might contribute to the risk stratification of a future ischemic event (42). Adding to that affirmative, Bos D et al., in a large population-based sample of asymptomatic individuals, associate IPH with new-onset stroke and cardiovascular disease (CVD), independent of other plaque characteristics (43). Patients with IPH are prone to develop two times more stroke or coronary heart disease (CHD) within 5 years. Their findings showed that even in asymptomatic individuals with low-grade stenosis, IPH is crucial for developing a first-ever stroke.

### Prediciton of Secondary Stroke

The majority of recurrences occur within 1 year and in the same anatomic region as the first stroke (44). Especially in patients with high-grade (70–99%) timely intervention can prevent recurrent ischemic stroke (45).

Two major trials, the European Carotid Surgery Trial (ECST) and the North American Symptomatic Carotid Endarterectomy Trial (NASCET), provided the vascular community in the early '90s with information regarding symptomatic patients. They showed that benefit from surgery was more significant in men than in women and in the elderly, and benefit decreased with time since the last symptoms. These observations were consistent across the 50% to 69%, and 70% to 99% stenosis groups and the two trials. These subgroup observations were sufficiently robust to guide the use of carotid endarterectomy (CEA) in routine clinical practice (46, 47).

Schindler A *et al*. showed an HRs of 10.2 for future ipsilateral stroke patients with symptomatic stenosis and 7.9 in patients with asymptomatic stenosis when IPH was characterized. In symptomatic patients, IPH demonstrated an increased risk of stroke at any degree of stenosis, even among patients with plaques <50% of stenosis (13).

With improved imaging methods, identifying patients with a higher risk of stroke may benefit the selection for CEA, allowing surgery to be indicated in patients with the highest benefit. Based on observed HRs on multivariable Cox regression in recently symptomatic patients with carotid stenosis, Kelly PJ *et al*. derived the SCAIL (symptomatic carotid atheroma inflammation lumen-stenosis) score by assigning points based on ^18^F-fluorodeoxyglucose (^18^F-FDG) uptake and stenosis severity, further discussed (48).

### Latest Studies Result on Vulnerable Carotid Plaques

Current studies and research groups aim to establish a relation between plaque vulnerability biomarkers, showing that identifying vulnerable carotid plaques with MRI helps predict ischemic stroke (Table 1).

**Table 1:** Imaging studies analyzing carotid plaque components and morphology on cerebrovascular risk in symptomatic and asymptomatic patients.

**Study**	**Imaging method**	**Variable analyzed**	**Central conclusion**
Rotterdam Study	MRI	IPH, LRNC	The size of IPH and not the presence of a lipid core was associated with symptomatic plaques in patients with recent ischemic event
ARIC Study	MRI	PT, LRNC	The presence of a lipid core was independently associated with incident CVD events when adjusted for traditional CVD risk factors and maximum CA wall thickness
Magnetic Study	MRI	IPH, FC, LRNC	Plaque composition in patients on treatment for asymptomatic carotid atherosclerosis shows no correlation between plaque vulnerability and the most well-controlled modifiable RF. Optimized therapy might have altered the association.
PARISK Study	MRI	IPH	The association between Lp(a) concentration and IPH supports the hypothesis that Lp(a) has a role in the process of atherosclerosis
CAPIAS Study	MRI	IPH, FC, LRNC	IPH, a ruptured FC, or the presence of a mural thrombus, was more frequent ipsilateral to ischemic stroke compared with that of the contralateral side
BIOVASC	PET/CT	^18^F-FDG uptake	Plaque ^18^F-FDG uptake was associated with early recurrent stroke in patients with recently symptomatic carotid stenosis.

*MRI, Magnetic Resonance Imaging; US, Ultrasound; IPH, intraplaque haemorrhage; LRNC, lipid-rich necrotic core; PT, plaque thickness; FC, fibrous cap; RF, risk factor; Lp, Lipoprotein; ^18^F-FDG, ^18^F-fluorodeoxyglucose*.

MRI currently provides the most proper imaging technique to specify lumen stenosis and features of plaque vulnerability, accurately assessing LRNC, FC, IPH, calcification, and plaque surface. This imaging technique has been used in almost every trial (49).

In the Rotterdam study, Van den Bouwhuijsen *et al*. showed a correlation between hypertension, current smoking, and presence of IPH and between hypercholesterolemia and LRNC in asymptomatic patients (50). They also showed a correlation of IPH and cortical infarcts. The size of IPH and not the presence of a lipid core was associated with symptomatic plaques in patients with recent ischemic event (51).

The ARIC Study also observed that carotid artery plaque burden and plaque eccentricity measures were directly associated with atherogenic cholesterol content. Their results suggested that measures of plaque burden could be use to control disease progression during the usage of therapies that lower atherogenic lipids. In contrast, LRNC could be an interesting imaging feature to follow (52).

Wasserman *et al*. previously already observed in the first study of associations between plaque lipid core and cardiovascular risk factors, the MESA study, a strong association with plasma cholesterol but not with hypertension, smoking, diabetes, or inflammatory factors, deducing that non-High-density lipoprotein cholesterol (non-HDL-C), which includes low-density lipoprotein (LDL) and Lipoproteins (Lp), may be of prime importance for the development of the clinically significant lipid-rich atherosclerotic plaque (53).

The MAGNETIC Study found no association between vulnerable plaque features and a history of hypertension, diabetes mellitus, hypercholesterolemia, and smoking. The authors speculate that optimizing medical therapy and a healthier lifestyle might have altered the association between plaque vulnerability and risk factors. A critical limit in this study concerns the absence of information on how long patients have been receiving treatment on atherosclerosis at the enrollment. This information could be used for a better understanding of medical therapy impact and lifestyle on plaque vulnerability (54).

The same rationale of a progression from vulnerable plaques to more stable plaques, both in carotid arteries, has been recently observed in the HeCES Study, published by Nuotio *et al*. In 10 years follow up, plaques related to ischemic event reveal more fibrous and non-inflammatory characteristics when compared to the initial features, due to lifestyle changes and effects of statins (55).

In the PARISK study, in patients with recent TIA or minor ischemic stroke, novel associations between (Lp)(a), concentrations and plaque features were identified. In women, elevated plasma Lp(a) levels were associated with higher prevalence of IPH and in men, elevated Lp(a) levels were associated with a higher degree of stenosis. The association between Lp(a) concentration and these vulnerable plaque characteristics supports the hypothesis that Lp(a) has a role in the process of atherosclerosis (56).

The CAPIAS prospective Study was a multicenter study with plaque imaging obtained within 10 days after symptom onset. The most frequent feature of ipsilateral carotid plaque was IPH. Ipsilateral LRNCs, a feature that is not part of the American Heart Association–lesion type definition, were larger in cryptogenic stroke compared with the reference group (57).

In a recently published cohort study by Kelly PJ *et al*., the Biomarkers/Imaging Vulnerable Atherosclerosis in Symptomatic Carotid disease Study (BIOVASC), patients with carotid stenosis and recent stroke/(TIA) were followed up, being the primary outcome any non-procedural ipsilateral recurrent stroke within 90 days of the index stroke/TIA. One hundred and nine patients were recruited into BIOVASC and had positron emission tomography (PET)/CT completed. In patients with recently symptomatic carotid stenosis. Plaque ^18^F-FDG uptake was associated with early recurrent stroke in patients with recently symptomatic carotid stenosis. They showed for the first time that plaque FDG uptake independently predicts early stroke after PET. This finding suggests that higher plaque FDG uptake is a marker of vulnerable carotid plaque leading to stroke recurrence and not a secondary consequence of recurrent brain infarction (58). These results led to a novel score called SCAIL Score.

### Symptomatic Carotid Atheroma Inflammation Lumen Stenosis Score (SCAIL)

This recently published model for estimating the risk of recurrent ischemic stroke included ^18^F-FDG standardized uptake values on PET-CT as a parameter for plaque inflammation. (48) Uptake of radiolabeled ^18^F-FDG on PET is a validated marker for plaque metabolism caused by inflammation and is associated with markers of plaque instability and late clinical events (59).

This model categorized ^18^F-FDG uptake and stenosis. On multivariable analysis, the SCAIL score independently predicted recurrent stroke after PET imaging, in addition to its association with all recurrent stroke events before or after PET. This suggests that early ^18^F-FDG-PET after hospital presentation may have prognostic utility to identify high-risk patients with carotid stenosis. It was shown for the first time that incorporating information relating to plaque inflammation-related metabolism and lumen stenosis in a single measure identifies patients at the highest risk of early recurrent stroke.

## Perspectives

The big challenge remains in defining, among the many plaque features, those that are pivotal for the optimized treatment. Big imaging data acquisition associated with artificial intelligence analysis is leading this field of research to a higher level.

The development of a score system based on imaging features of plaque vulnerability may provide clinicians with a better tool to approach the disease.

## Author Contributions

MG and RF contributed to the conception and design of the review. MG, RF, MS, and CF wrote the first draft of the manuscript. MG, RF, MS, WA, CF, SS, and AG wrote sections of the manuscript. All authors contributed to manuscript revision, read, and approved the submitted version.

## Conflict of Interest

The authors declare that the research was conducted in the absence of any commercial or financial relationships that could be construed as a potential conflict of interest.

## Publisher's Note

All claims expressed in this article are solely those of the authors and do not necessarily represent those of their affiliated organizations, or those of the publisher, the editors and the reviewers. Any product that may be evaluated in this article, or claim that may be made by its manufacturer, is not guaranteed or endorsed by the publisher.

## References

[1] Aboyans V Ricco JB Bartelink MLEL Björck M Brodmann M Cohnert T. 2017 ESC guidelines on the diagnosis and treatment of peripheral arterial diseases, in collaboration with the European Society for Vascular Surgery (ESVS). Eur Heart J. (2018) 39:763–816. 10.1093/eurheartj/ehx095 29425606

[2] Eliasziw M Rankin RN Fox AJ Haynes RB Barnett HJM. Accuracy and prognostic consequences of ultrasonography in identifying severe carotid artery stenosis. Stroke. (1995) 26:1747–52. 10.1161/01.STR.26.10.1747 7570719

[3] Flumignan CDQ Flumignan RLG Navarro TP. Extracranial carotid stenosis: evidence based review. Rev Col Bras Cir. (2017) 44:293–301. 10.1590/0100-69912017003012 28767806

[4] Saba L Saam T Jäger HR Yuan C Hatsukami TS Saloner D. Imaging biomarkers of vulnerable carotid plaques for stroke risk prediction and their potential clinical implications. Lancet Neurol. (2019) 18:559–72. 10.1016/S1474-4422(19)30035-3 30954372

[5] Mahabadi AA Rassaf T. Imaging of coronary inflammation for cardiovascular risk prediction. Lancet. (2018) 392:894–6. 10.1016/S0140-6736(18)31716-1 30170850

[6] Kerwin WS Miller Z Yuan C. Imaging of the high-risk carotid plaque: magnetic resonance imaging. Semin Vasc Surg. (2017) 30:54–61. 10.1053/j.semvascsurg.2017.04.009 28818259

[7] Michel JB Martin-Ventura JL Nicoletti A Ho-Tin-Noé B. Pathology of human plaque vulnerability: Mechanisms and consequences of intraplaque haemorrhages. Atherosclerosis. (2014) 234:311–9. 10.1016/j.atherosclerosis.2014.03.020 24726899

[8] Kolodgie FD Yahagi K Mori H Romero ME Trout HH Finn AV. High-risk carotid plaque: lessons learned from histopathology. Semin Vasc Surg. (2017) 30:31–43. 10.1053/j.semvascsurg.2017.04.008 28818257

[9] Le Dall J Ho-Tin-Noe B Louedec L Meilhac O Roncal C Carmeliet P. Immaturity of microvessels in haemorrhagic plaques is associated with proteolytic degradation of angiogenic factors. Cardiovasc Res. (2010) 85:184–93. 10.1093/cvr/cvp253 19620132

[10] Dilba K van Dijk AC Crombag GAJC van der Steen AFW Daemen MJ Koudstaal PJ. Association between intraplaque hemorrhage and vascular remodeling in carotid arteries: the Plaque at RISK (PARISK) study. Cerebrovasc Dis. (2021) 50:94–9. 10.1159/000511935 33271533

[11] Deng F Mu C Yang L Li H Xiang X Li K. Carotid plaque magnetic resonance imaging and recurrent stroke risk: a systematic review and meta-analysis. Medicine (Baltimore). (2020) 99:e19377. 10.1097/MD.0000000000019377 32221065 PMC7220511

[12] Kamtchum-Tatuene J Wilman A Saqqur M Shuaib A Jickling GC. Carotid plaque with high-risk features in embolic stroke of undetermined source: systematic review and meta-analysis. Stroke. (2020) 51:311–4. 10.1161/STROKEAHA.119.027272 31752616 PMC6993880

[13] Schindler A Schinner R Altaf N Hosseini AA Simpson RJ Esposito-Bauer L. Prediction of stroke risk by detection of hemorrhage in carotid plaques. JACC Cardiovasc Imaging. (2020) 13:395–406. 10.1016/j.jcmg.2019.03.028 31202755

[14] McNally JS Kim SE Mendes J Hadley JR Sakata A De Havenon AH. Magnetic resonance imaging detection of intraplaque hemorrhage. Magn Reson Insights. (2017) 10:1178623X1769415. 10.1177/1178623X17694150 28469441 PMC5348123

[15] Altaf N Daniels L Morgan PS Auer D MacSweeney ST Moody AR. Detection of intraplaque hemorrhage by magnetic resonance imaging in symptomatic patients with mild to moderate carotid stenosis predicts recurrent neurological events. J Vasc Surg. (2008) 47:337–42. 10.1016/j.jvs.2007.09.064 18164171

[16] Qiao Y Etesami M Malhotra S Astor BC Virmani R Kolodgie FD. Identification of intraplaque hemorrhage on MR angiography images: a comparison of contrast-enhanced mask and time-of-flight techniques. Am J Neuroradiol. (2011) 32:454–9. 10.3174/ajnr.A2320 21233234 PMC3337083

[17] Boodt N Compagne KCJ Dutra BG Samuels N Tolhuisen ML Alves HCBR. Stroke etiology and thrombus computed tomography characteristics in patients with acute ischemic stroke: a MR CLEAN registry substudy. Stroke. (2020) 51:1727–35. 10.1161/STROKEAHA.119.027749 32404040

[18] Daghem M Bing R Fayad ZA Dweck MR. Noninvasive imaging to assess atherosclerotic plaque composition and disease activity. JACC Cardiovasc Imaging. (2020) 13:1055–68. 10.1016/j.jcmg.2019.03.033 31422147 PMC10661368

[19] Shi X Han Y Li M Yin Q Liu R Wang F. Superficial calcification with rotund shape is associated with carotid plaque rupture: an optical coherence tomography study. Front Neurol. (2020) 11:563334. 10.3389/fneur.2020.563334 33071946 PMC7530839

[20] Kolodgie FD Burke AP Farb A Gold HK Yuan J Narula J.. The thin-cap fibroatheroma: a type of vulnerable plaque: the major precursor lesion to acute coronary syndromes. Curr Opin Cardiol. (2001) 16:285–92. 10.1097/00001573-200109000-00006 11584167

[21] Hjelmgren O Johansson L Prahl U Schmidt C Fredén-Lindqvist J Bergström GML. study of plaque vascularization and inflammation using quantitative contrast-enhanced US and PET/CT. Eur J Radiol. (2014) 83:1184–9. 10.1016/j.ejrad.2014.03.021 24767629

[22] Fedak A Chrzan R Chukwu O Urbanik A. Ultrasound methods of imaging atherosclerotic plaque in carotid arteries: examinations using contrast agents. J Ultrason. (2020) 20:191–200. 10.15557/JoU.2020.0032 33365156 PMC7705485

[23] Fedak A Ciuk K Urbanik A. Ultrasonography of vulnerable atherosclerotic plaque in the carotid arteries: B-mode imaging. J Ultrason. (2020) 20:e135–45. 10.15557/JoU.2020.0022 32609972 PMC7418858

[24] Moguillansky D Leng X Carson A Lavery L Schwartz A Chen X. Quantification of plaque neovascularization using contrast ultrasound: a histologic validation. Eur Heart J. (2011) 32:646–53. 10.1093/eurheartj/ehq197 20581005 PMC3046374

[25] Kerwin W Hooker A Spilker M Vicini P Ferguson M Hatsukami T. Quantitative magnetic resonance imaging analysis of neovasculature volume in carotid atherosclerotic plaque. Circulation. (2003) 107:851–6. 10.1161/01.CIR.0000048145.52309.31 12591755

[26] van den Oord SCH. ten Kate GL, Sijbrands EJG, van der Steen AFW, Schinkel AFL. Effect of carotid plaque screening using contrast-enhanced ultrasound on cardiovascular risk stratification. Am J Cardiol. (2013) 111:754–9. 10.1016/j.amjcard.2012.11.033 23266072

[27] Saito H Kuroda S Hirata K Magota K Shiga T Tamaki N. Validity of dual MRI and ^18^ F-FDG PET imaging in predicting vulnerable and inflamed carotid plaque. Cerebrovasc Dis. (2013) 35:370–7. 10.1159/000348846 23635390

[28] Tawakol A Migrino RQ Bashian GG Bedri S Vermylen D Cury RC. *In Vivo* 18F-fluorodeoxyglucose positron emission tomography imaging provides a noninvasive measure of carotid plaque inflammation in patients. J Am Coll Cardiol. (2006) 48:1818–24. 10.1016/j.jacc.2006.05.076 17084256

[29] Derlin T Richter U Bannas P Begemann P Buchert R Mester J. Feasibility of ^18^ F-Sodium Fluoride PET/CT for imaging of atherosclerotic plaque. J Nucl Med. (2010) 51:862–5. 10.2967/jnumed.110.076471 20484438

[30] Skagen K Johnsrud K Evensen K Scott H Krohg-Sørensen K Reier-Nilsen F. Carotid plaque inflammation assessed with ^18^ F-FDG PET/CT is higher in symptomatic compared with asymptomatic patients. Int J Stroke. (2015) 10:730–6. 10.1111/ijs.12430 25588553

[31] Fujimoto K Norikane T Yamamoto Y Takami Y Mitamura K Okada M. Association between carotid 18F-NaF and 18F-FDG uptake on PET/CT with ischemic vascular brain disease on MRI in patients with carotid artery disease. Ann Nucl Med. (2019) 33:907–15. 10.1007/s12149-019-01403-3 31571042

[32] Tardif JC Lesage F Harel F Romeo P Pressacco J. Imaging biomarkers in atherosclerosis trials. Circ Cardiovasc Imaging. (2011) 4:319–33. 10.1161/CIRCIMAGING.110.962001 21586743

[33] Zhao X Underhill HR Zhao Q Cai J Li F Oikawa M.. Discriminating carotid atherosclerotic lesion severity by luminal stenosis and plaque burden: a comparison utilizing high-resolution magnetic resonance imaging at 30 tesla. Stroke. (2011) 42:347–53. 10.1161/STROKEAHA.110.597328 21183749 PMC5542669

[34] Glagov S Weisenberg E Zarins CK Stankunavicius R Kolettis GJ. Compensatory enlargement of human atherosclerotic coronary arteries. N Engl J Med. (1987) 316:1371–5. 10.1056/NEJM198705283162204 3574413

[35] Ball S Rogers S Kanesalingam K Taylor R Katsogridakis E McCollum C. Carotid plaque volume in patients undergoing carotid endarterectomy. Br J Surg. (2018) 105:262–9. 10.1002/bjs.10670 29315509 PMC5873399

[36] Rafailidis V Chryssogonidis I Tegos T Kouskouras K Charitanti-Kouridou A. Imaging of the ulcerated carotid atherosclerotic plaque: a review of the literature. Insights Imaging. (2017) 8:213–25. 10.1007/s13244-017-0543-8 28160261 PMC5359146

[37] Li J Li D Yang D Hang H Wu Y Yao R. Irregularity of carotid plaque surface predicts subsequent vascular event: a MRI study. J Magn Reson Imaging. (2020) 52:185–94. 10.1002/jmri.27038 31944452

[38] Homburg PJ Rozie S van Gils MJ van den Bouwhuijsen QJA Niessen WJ Dippel DWJ. Association between carotid artery plaque ulceration and plaque composition evaluated with multidetector CT angiography. Stroke. (2011) 42:367–72. 10.1161/STROKEAHA.110.597369 21183745

[39] Hamada O Sakata N Ogata T Shimada H Inoue T. Contrast-enhanced ultrasonography for detecting histological carotid plaque rupture: quantitative analysis of ulcer. Int J Stroke. (2016) 11:791–8. 10.1177/1747493016641964 27256473

[40] Bos D van Dam-Nolen DHK Gupta A Saba L Saloner D Wasserman BA. Advances in multimodality carotid plaque imaging: *AJR* expert panel narrative review. Am J Roentgenol. (2021) 217:16–26. 10.2214/AJR.20.24869 33438455

[41] Saam T Hetterich H Hoffmann V Yuan C Dichgans M Poppert H. Meta-Analysis and systematic review of the predictive value of carotid plaque hemorrhage on cerebrovascular events by magnetic resonance imaging. J Am Coll Cardiol. (2013) 62:1081–91. 10.1016/j.jacc.2013.06.015 23850912

[42] Kurosaki Y Yoshida K Fukuda H Handa A Chin M Yamagata S. Asymptomatic carotid T1-high-intense plaque as a risk factor for a subsequent cerebrovascular ischemic event. Cerebrovasc Dis. (2017) 43:250–6. 10.1159/000455973 28259876

[43] Bos D Arshi B van den Bouwhuijsen QJA Ikram MK Selwaness M Vernooij MW. Atherosclerotic carotid plaque composition and incident stroke and coronary events. J Am Coll Cardiol. (2021) 77:1426–35. 10.1016/j.jacc.2021.01.038 33736825

[44] Sacco RL Wolf PA Kannel WB McNamara PM. Survival and recurrence following stroke. the framingham study. Stroke. (1982) 13:290–5. 10.1161/01.STR.13.3.290 7080120

[45] Orrapin S Rerkasem K. Carotid endarterectomy for symptomatic carotid stenosis. Cochrane Database Syst Rev. (2017) 2017:CD001081. 10.1002/14651858.CD001081.pub3 28590505 PMC6481587

[46] Randomised trial of endarterectomy for recently symptomatic carotid stenosis: final results of the MRC European Carotid Surgery Trial (ECST). Lancet Lond Engl. (1998) 351:1379–87. 10.1016/S0140-6736(97)09292-1 9593407

[47] Paciaroni M Eliasziw M Kappelle LJ Finan JW Ferguson GG Barnett HJ. Medical complications associated with carotid endarterectomy. North American Symptomatic Carotid Endarterectomy Trial (NASCET). Stroke. (1999) 30:1759–63. 10.1161/01.STR.30.9.1759 10471420

[48] Kelly PJ Camps-Renom P Giannotti N Martí-Fàbregas J McNulty JP Baron JC. A risk score including carotid plaque inflammation and stenosis severity improves identification of recurrent. Stroke. (2020) 51:838–45. 10.1161/STROKEAHA.119.027268 31948355

[49] Porambo ME DeMarco JK MR. imaging of vulnerable carotid plaque. Cardiovasc Diagn Ther. (2020) 10:1019–31. 10.21037/cdt.2020.03.12 32968658 PMC7487401

[50] van den Bouwhuijsen QJA Vernooij MW Verhaaren BFJ Vrooman HA Niessen WJ Krestin GP. Carotid plaque morphology and ischemic vascular brain disease on MRI. Am J Neuroradiol. (2017) 38:1776–82. 10.3174/ajnr.A5288 28705824 PMC7963699

[51] Ikram MA van der Lugt A Niessen WJ Koudstaal PJ Krestin GP Hofman A. The Rotterdam Scan Study: design update 2016 and main findings. Eur J Epidemiol. (2015) 30:1299–315. 10.1007/s10654-015-0105-7 26650042 PMC4690838

[52] Virani SS Catellier DJ Pompeii LA Nambi V Hoogeveen RC Wasserman BA. Relation of cholesterol and lipoprotein parameters with carotid artery plaque characteristics: the Atherosclerosis Risk in Communities (ARIC) carotid MRI study. Atherosclerosis. (2011) 219:596–602. 10.1016/j.atherosclerosis.2011.08.001 21868017 PMC3226845

[53] Wasserman BA Sharrett AR Lai S Gomes AS Cushman M Folsom AR. Risk factor associations with the presence of a lipid core in carotid plaque of asymptomatic individuals using high-resolution MRI: the Multi-Ethnic Study of Atherosclerosis (MESA). Stroke. (2008) 39:329–35. 10.1161/STROKEAHA.107.498634 18174475

[54] Catalano O Bendotti G Mori A De Salvo M Falconi M Aloi TL. Evolving determinants of carotid atherosclerosis vulnerability in asymptomatic patients from the MAGNETIC observational study. Sci Rep. (2021) 11:2327. 10.1038/s41598-021-81247-y 33504842 PMC7840938

[55] Nuotio K Ijäs P Heikkilä HM Koskinen SM Saksi J Vikatmaa P. Morphology and histology of silent and symptom-causing atherosclerotic carotid plaques – rationale and design of the Helsinki Carotid Endarterectomy Study 2 (the HeCES2). Ann Med. (2018) 50:501–10. 10.1080/07853890.2018.1494851 30010425

[56] van Dam-Nolen DHK van Dijk AC Crombag GAJC Lucci C Kooi ME Hendrikse J. Lipoprotein(a) levels and atherosclerotic plaque characteristics in the carotid artery: the Plaque at RISK (PARISK) study. Atherosclerosis. (2021) 329:22–9. 10.1016/j.atherosclerosis.2021.06.004 34216874

[57] Kopczak A Schindler A Bayer-Karpinska A Koch ML Sepp D Zeller J. Complicated carotid artery plaques as a cause of cryptogenic stroke. J Am Coll Cardiol. (2020) 76:2212–22. 10.1016/j.jacc.2020.09.532 33153580

[58] Kelly PJ Camps-Renom P Giannotti N Martí-Fàbregas J Murphy S McNulty J. Carotid plaque inflammation imaged by ^18^ F-Fluorodeoxyglucose positron emission tomography and risk of early recurrent stroke. Stroke. (2019) 50:1766–73. 10.1161/STROKEAHA.119.025422 31167623

[59] Masteling MG Zeebregts CJ Tio RA Breek JC Tietge UJF de Boer JF. High-resolution imaging of human atherosclerotic carotid plaques with micro18F-FDG PET scanning exploring plaque vulnerability. J Nucl Cardiol. (2011) 18:1066–75. 10.1007/s12350-011-9460-2 22002650 PMC3225624

