# Supplementary material for: Carotid Plaque Composition and the Importance of Non-Invasive in Imaging Stroke Prevention
Source: Front Cardiovasc Med. 2022 May 16;9:885483. doi: 10.3389/fcvm.2022.885483 (PMC9149096; doi:10.3389/fcvm.2022.885483)
Supplement: Supplementary file 2 [file Data_Sheet_1.docx]

Supplementary Material

**Supplementary Material:**

The ultrasound video shows significant enhancement in the plaque (arrow) because of the presence of microbubble. In the ultrasound video, the two panels show the difference in sensitivity using conventional B-mode and microbubble injection
